# Trends in Pediatric and Adolescent Anterior Cruciate Ligament Injuries in Victoria, Australia 2005–2015

**DOI:** 10.3390/ijerph14060599

**Published:** 2017-06-05

**Authors:** Louise Shaw, Caroline F. Finch

**Affiliations:** Australian Collaboration for Research into Injury in Sport and its Prevention, Federation University Australia, Ballarat 3353, Victoria, Australia; louise.shaw@federation.edu.au

**Keywords:** sports injury, knee injury, ACL injury, pediatrics, adolescents

## Abstract

Anterior cruciate ligament (ACL) injuries in children and adolescents have been the focus of recent media attention and parental concern, given their potential for adverse long-term health outcomes and healthcare costs. However, there is limited formal evidence on trends in the incidence of ACL injuries in children. This study utilizes the Victorian Admitted Episodes Dataset (VAED) to characterize epidemiologic trends of hospital-admitted ACL injuries in those aged 5 to 14 years over a period of 10 years from 2005 to 2015. There was a total of 320 cases and the overall annual rate of ACL injuries increased by 147.8% from 2.74 per 100,000 population in 2005/2006 to 6.79 per 100,000 in 2014/2015. The majority (96.9%) of these injuries were in 10- to 14-year-olds. The main in-hospital procedure provided to over 80% of the hospitalized cases involved ACL reconstruction. Sporting activities accounted for 56.6% of ACL injuries. For females, over half (52.4%) of ACL injuries occurred whilst playing ball sports, compared to 35.4% of males. The large increase in ACL injuries in 5- to 14-year-olds in the state of Victoria, Australia over a 10-year period indicates they are a significant and emerging health burden. Population-wide ACL prevention policies are required to halt these trends. Cost effective prevention programs that involve neuromuscular training must be implemented in schools and junior sports teams.

## 1. Introduction

Children and adolescent participation in organized sports is widespread and gaining in popularity in Western countries [1], with children now often beginning participation at a younger age and competing at a higher level [2]. In addition, greater demands are being placed on youth athletes, through increased training, sports specialization, and an emphasis on year-round competitive play [2]. Taken together, these factors have led to an increase in the diagnosis of sports-specific injuries in children and adolescents [1,2,3]. Anterior Cruciate Ligament (ACL) injury to the knee, particularly in children and adolescents, has been the focus of recent media and scholarly attention in Australia [4,5]. This is not surprising given that ACL injury causes significant discomfort and disability and may also result in the long-term in reduced levels of physical activity and contribute to obesity [6], thus negating the potential benefits of sports participation for children and adolescents. In addition, ACL injuries have the potential for adverse long-term health outcomes, such as an increased risk of degenerative arthritis and escalation of health care costs [1,7,8].

Historically, ACL tears in the skeletally immature population were thought to be an uncommon injury [2]. For example, studies in the late 1970s and early 1980s reported the incidence of ACL rupture in skeletally immature individuals sustaining a knee injury as being between 1.0% and 3.4% [9,10,11,12]. However, as imaging and clinical awareness of injuries in young athletes has improved, the diagnosis and reported incidence of ACL injury in the skeletally immature population has increased [13], and ACL tears in pediatric and adolescent patients are no longer considered rare [7,14]. Whilst it is possible that improved methods of diagnosis are responsible for greater rates of reconstruction, or that reconstruction rates have increased due to improved methods of treatment in the skeletally immature, it is also possible that the incidence of ACL tears is truly increasing in children and adolescents [2]. Research from the USA on the incidence of ACL injuries in children and adolescents suggests an increased frequency in this population [7,15]. There is also emerging evidence to support this claim in Australia. A review of 212 arthroscopies performed in the Adelaide Children’s Hospital from November 1980 to June 1986, reported 31 cases (14.6%) of ACL pathology with an age range of 8 to 18 years, and 12 of these patients were aged less than 14 years old [16].

The rate of ACL injuries in children and adolescents does not appear to be uniform across age groups [17]. Data from a pediatric medical center in Boston, United States of America (USA) between 2000 and 2009 showed that ACL injuries account for 6.3% of all sports injuries in 5- to 12-year-olds, but 10.6% in 13- to 17-year-olds [18]. A review of ACL construction rates over 20 years from 1990 to 2009 in New York State found that the rate of ACL reconstruction was eight times more in 15- to 18-year-olds than in 11- to 14-year-olds [2]. Data obtained from a national database in the USA for ACL tears in pediatric and adolescent patients for those who underwent arthroscopic reconstruction between 2007 and 2011 showed that the most significant increases were in 10- to 14-year-olds with an 18.9% increase in the diagnosis of ACL tear (*p* < 0.0001) and a 26.7% increase in ACL reconstruction (*p* < 0.0001) [14]. A statistically significant increase was also noted in the diagnosis of ACL tears in the 5–9 year-old cohort (4.5% increase, *p* < 0.0001) [14]. Australian data on ACL reconstructions between July 2003 and June 2008 showed a significant linear increase for males in the age group of 5–14 years (*p* = 0.005) and also demonstrated that the incidence of ACL reconstruction rose rapidly through adolescence and early adulthood and then gradually declined [5]. These findings suggest that the ACL injury pattern coincides with the general injury pattern in children, that there is a low injury risk during middle childhood but this increases with age and participation in higher-level sports.

Epidemiological data on ACL injuries in skeletally immature athletes, particularly in Australia, and indeed elsewhere, are sparse [7]. The widespread anecdotal opinion is that ACL injury in children and adolescents in Australia is increasing [4]. There is an urgent need to monitor trends in ACL injury in this population in Australia to help guide policy development for prevention and for priority setting. The aim of this study was therefore to describe the trends in the incidence of all hospital-treated ACL injuries in Victoria in children and adolescents aged 5–14 years from July 2005 to June 2015.

## 2. Materials and Methods

The data analyzed for this study related to all Victorian public and private hospital admissions in the state of Victoria, Australia as reported in the routinely collected Victorian Admitted Episodes Dataset (VAED). De-identified, summary data were provided to the authors by the Victorian Injury Surveillance Unit (VISU), the repository for de-identified injury surveillance data in Victoria, Australia [19]. The VAED data for this period was coded to the International Statistical Classification of Diseases and Related Health Problems, Tenth Revision, Australian Modifications (ICD-10-AM) [20]. Data were selected on the basis that cases had a principal diagnosis of an injury (S00-T98) and occurred during the 10-year period covering the financial years from July 2005 to June 2015.

Data extraction was limited to those aged 5 to 14 years with an unintentional injury resulting in a hospital admission. Sports participation data in Australia classes those aged 5 to 14 years as children, and those 15 years and above as adults [21]. To ensure that there were comparable denominator data for injury rate estimates, analysis was limited to this age group. Cases were selected if the principal diagnosis was related to an ACL-related injury, specifically the following ICD-10-AM codes: 23.51: Chronic instability of the knee-ACL; 23.61: Other spontaneous disruption of ligaments of the knee-ACL; 23.81: Other internal derangements of knee-ACL; 23.91: Internal derangement of knee unspecified-ACL; S83.51: Sprain and strain of the knee; S83.53: Rupture of ACL. In the ICD-10-AM, activity codes in the range of U50-U71 indicate that the injury occurred during sport or active recreation. Data were extracted from the VAED on the patient age, sex, principal injury diagnosis, activity at the time of injury, first listed procedure code classified according to the Australian Classification of Health Interventions (ACHI) [20], the financial year of presentation, and length of stay in hospital. Admissions as a result of transfer from another hospital or due to a statistical separation from the same hospital were excluded to minimize double counting of incident cases. Annual population data and numbers were extracted from the Australian Bureau of Statistics (ABS) [22] on the age and sex of the population for the state of Victoria.

Injury rates were calculated using the specific number of incident cases injuries in each financial year as the numerator, and the estimated population for each year (calculated from the ABS) as the denominator. The rates were then expressed as a number per 100,000 population. The population injury rates and 95% confidence intervals (CIs) were calculated with the Mid-P exact test using Miettinen’s modification [23].

## 3. Results

Between July 2005 and June 2015 inclusive, there were 320 hospital-treated ACL injuries in children aged 5 to 14 years in Victoria. This included the following age group totals: 5- to 9-year-olds, 10 diagnoses (3.1%); and 10- to 14-year-olds, 310 diagnoses (96.9%). Over half (*n* = 175, 54.7%) of those injured were male and the remainder were female (*n* = 145, 45.3%). The principal diagnosis for the majority (*n* = 282, 88.1%) of children and adolescents was rupture of the ACL (ICD-10-AM 583.53).

**Figure 1:**
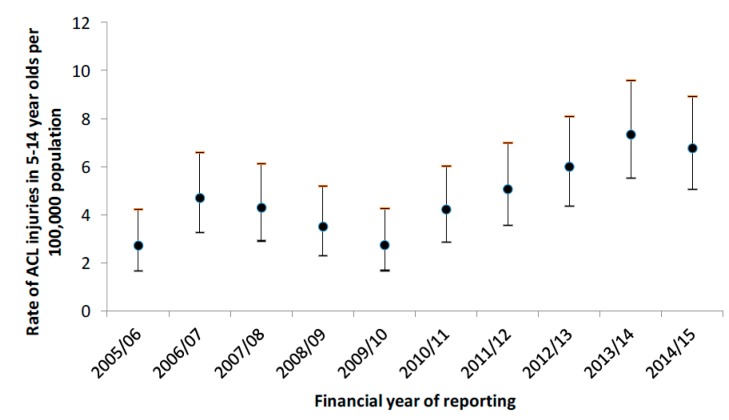
Person time rates per 100,000 population for ACL injuries admitted to hospital in Victoria amongst 5- to 14-year-olds with 95% confidence intervals.

Figure 1 shows the population-adjusted injury rates and 95% CIs for hospital admissions for ACL injuries in adolescents aged 5 to 14 years in Victoria, from July 2005 to June 2015. The incidence rate of ACL injuries in children and adolescents increased by 147.8% from 2.74 per 100,000 population in 2005/2006 to 6.79 per 100,000 population in 2014/2015.

Table 1 shows the classification of the first listed in-hospital procedure/treatment provided to the admitted ACL injuries amongst Victorian children and adolescents from 2005 to 2015. Over 80% of the health interventions involved reconstruction of the knee or ACL.

Over half (*n* = 181, 56.6%) of the hospitalized ACL injuries were coded as having occurred during sporting activities. A further 40.3% (*n* = 129) were during other activity (unspecified). This corresponds to 94.8% of all cases with a known activity at the time of injury, being injured during sport or recreation. Table 2 summarizes the type of activity being undertaken when the ACL injury occurred for males and females. Amongst females, over half (52.4%) injured their ACL whilst playing team ball sports, whereas for males, almost half (47.4%) were taking part in other activity (unspecified). Where cell values were less than five, the values were suppressed.

## 4. Discussion

This study is the first to describe trends in ACL injuries in children and adolescents in Victoria, Australia, based on acute care admissions to hospitals. The incidence of hospital-treated ACL injuries in people aged 5 to 14 years increased by 147.8% in Victoria, Australia between July 2005 and June 2015.

These findings support those of studies in other countries, such as the USA, that the rate of ACL injuries in pediatrics and adolescents is increasing [2,7,14,24]. Previous research has reported an increase in the rate of hospital-treated sports injuries in children in the same geographic region over the same period [25]. Based on our findings, ACL injuries in pediatric and adolescent patients should no longer be considered a rare injury. Compared with the increase in ACL tear diagnosis in adult patients between 2007 and 2011, injury in pediatric and adolescent groups seems to be increasing at a higher rate [14]. This increase in ACL injuries in children and adolescents could potentially place a substantial burden on the health delivery system of Victoria [26]. Furthermore, an increase in ACL injuries in children and adolescents means that more people are at risk of developing future health problems, such as osteoarthritis, as a result of such injuries [26]. With more children and adolescents being encouraged to take up an active lifestyle, there is the potential for the rate of ACL injuries in this group to increase even further [26]. These findings add further strength to conclusions that sports injuries and specifically ACL injuries in children and adolescents are a population health issue, should be a priority for government health agencies, and necessitate the setting of sports injury prevention policies [25].

The literature is limited in relation to causes behind the increased rate of ACL tears in pediatric and adolescent athletes [27], however, they are likely to be multifactorial. The vast majority are sports injuries and contributing factors to their causation could include a rise in competitive sport activity, increased single sport concentration, year-round participation, and consequently less time in free, unstructured play [3,7,14,28]. A study of the anatomical risk factors for ACL injury has found that ACL-injured children have a narrower intercondylar notch and an increased medial or lateral tibial slope [29]. Further, sensorimotor function is not fully mature by the time children reach adolescence [30] and deficits in a variety of sensorimotor mechanisms have been correlated with increased ACL injury risk [31]. During rapid growth spurts, core strength, neuromuscular ability, and proprioception may be imbalanced and contribute to an increased risk of ACL tears [32]. New literature on superior outcomes for ACL tears with reconstruction over conservative management in pediatrics and adolescents [15,33,34,35,36], may have contributed to the increased diagnosis and repair of ACL tears.

The frequency and circumstances of ACL injuries also differs according to gender. Whilst in this study more males (54.7%) were hospitalized with ACL injuries than females (45.3%), over half (52.4%) of the females injured their ACL whilst playing team ball sports, compared to 35.4% of males. The higher level of unspecified activity at the time of injury in males could partially explain this difference. Previous studies have observed that prior to puberty there are no sex-related differences in ACL risk factors [17,27]. However, the rate of ACL injury has been found to be higher in female adolescents compared to their male counterparts, with sex disparity in injury rates attributed to sex-specific differences elicited at the onset of puberty and maturation [27,37]. A review of data from the National High School Sports-Related Injury Surveillance Study in the USA demonstrated that female high school athletes (aged 14 to 18 years) have higher rates of ACL injury compared with male athletes in the sports of baseball, softball, soccer, and basketball [7]. In female soccer players, the ACL injury rate in Sweden was found to be more than two-fold higher than their male counterparts, with adolescent females (aged 12 to 17 years) particularly at risk [38].

Neuromuscular control of the knee and landing forces has been found to be significantly worse in females than in males during the transition from pre-pubertal to pubertal stages, with females showing regressions in control abilities [32,37,39]. Adolescent female athletes are therefore at higher risk of ACL injury if preventive measures are not taken.

Given the increase in ACL injuries in children and adolescents, and the serious impact of ACL injury on young athletes, it is clear that prevention of ACL injuries is of great importance [1,26]. Trends could be reversed in the future through the implementation of validated injury prevention programs in community sports groups at the individual, group, and organizational level [40,41]. Programs with good technique and age-appropriate motor skills are vital. Multiple randomized controlled trials over the last two decades have demonstrated that ACL injury prevention through regular neuromuscular agility training programs is highly successful in the adolescent population [42,43,44,45]. Essential components of prevention programs in children and adolescents include strengthening, plyometric and balance training, and neuromuscular training with feedback to modify technique [46]. This may artificially induce the neuromuscular spurt and have the potential to reduce the risk of ACL injury in young athletes [46,47]. It is therefore recommended that young athletes be encouraged to partake in preseason training programs focused on strengthening, neuromuscular, and proprioceptive training, under the appropriate supervision of qualified personnel [46]. Given the increasing rate of ACL injury in Australian children and adolescents, and the demonstrated effectiveness of prevention programs, a national ACL injury prevention program for youth could improve health outcomes while simultaneously reducing medical costs [48]. However, whilst ACL injury prevention using neuromuscular training has been found to be successful in the adolescent population, the level of evidence for ACL prevention in those below the age of 12 years, is markedly lower [17] and therefore requires further research.

It is widely recognized that ACL prevention programs are of particular importance in female adolescent athletes, and recent studies have suggested that exercise prevention programs may reduce the risk of ACL injury in adolescent female athletes [7]. Neuromuscular training has demonstrated approximately a 50% reduction in ACL injury risk for female athletes [27,42,45]. A systematic review of studies of ACL prevention programs in female athletes under the age of 19 years found that only three ACL intervention programs (Sportsmetrics, Prevent Injury and Enhance Performance, and Knee Injury Prevention) successfully reduced non-contact ACL injury incidence rates in female adolescents [49]. High compliance with a 15 min warm-up neuromuscular training program in female Swedish soccer players aged 12 to 17 years resulted in an 88% reduction in the rates of ACL injury compared with players with low compliance [50]. A program that is composed of plyometrics, strength, flexibility, and agility exercises and supervised by trained instructors is required to achieve the desired changes in neuromuscular indices [27,49]. There appears to be little research that details ACL prevention programs and their effects on young children. ACL prevention programs for younger children would need to be appropriately tailored to their abilities and stage of musculoskeletal development. Given the increase in ACL injuries in this age group, such programs should be developed as a matter of priority.

Community-based injury prevention programs with excellent efficacy may however suffer very low levels of compliance, dissemination, and adoption if no consideration is given to how they can be properly implemented [51,52]. If ACL injury prevention strategies are to successfully reduce the population incidence of ACL injuries, co-operation between researchers, clinicians, and policy-makers is vital [53,54,55,56].

This study is the first population-based study of trends in acute ACL injuries in children and adolescents in Australia. The VAED encompasses every hospital (both private and public) in the state of Victoria, allowing a comprehensive and complete population-based dataset to determine the rate of ACL injury admissions. However, the study does have its limitations. The study relies on the accuracy of available data and, despite the VAED coding being completed by specialist coders, there are issues with the completeness and accuracy of the activity at time of injury (i.e., the sports injury) coding [57]. Furthermore, given the limitations to the routinely collected sports injury data, as has been noted elsewhere for this data, it was not possible to determine if the ACL injuries arose through highly competitive or high-intensity sport, informal sport, or more recreational forms of these activities [25]. It is also possible that some of the reported ACL injuries are re-injuries due to the de-identified nature of the data.

To effectively target prevention strategies to groups at high risk for ACL injuries without discouraging participation, participation rates and exposure data need to be collected and monitored [25]. As the total population of children in Victoria aged 5 to 14 years was used as the denominator for this study, our rate estimates do not provide information about relative exposure risks. It was not possible to calculate participation-adjusted injury rates due to the lack of annual population-level figures for child participation in sport. Nonetheless, the fact that the population of 5- to 14-year-olds increased by 7.4% over the 10-year period, compared with the 147.8% increase in hospitalized ACL tears, is highly suggestive that increased ACL injury frequency and rates are not solely due to changing demographics [25]. Compared to Boston, USA where the reported rate of increase in ACL injuries between 2000 and 2009 was only 6.3% [18], the much higher rate of increase in Australia is particularly concerning. The reasons for this difference are unknown, but may reflect different treatment priorities and actions in the two countries. Additionally, the nature of the risks in sports played in Australia compared to the USA may differ. Both of these reasons are worthy of future research.

The dataset presented in this paper is likely to be an underestimation of all pediatric and adolescent ACL injuries, as it only includes cases that were admitted to hospital. Many cases of sports injuries are likely to have sought treatment from a General Practitioner (doctor) or other musculoskeletal expert such as a physiotherapist [58,59]. It is also therefore unclear whether the increase in the population-adjusted hospitalized ACL injury rates seen in this study is largely limited to ACL injuries that require hospital treatment, or if there has also an increase in ACL injuries that do not require hospital treatment because of the lack of available population-level data. Further population-based studies are required to examine the incidence and impact of ACL injuries not treated in the hospital setting. Increased awareness of the potential for ACL tears in skeletally immature patients and more aggressive diagnosis and evaluation with Magnetic Resonance Imaging (MRI) are also likely to contribute to the increase, [14] and more definitive studies would help clarify this.

Finally, the data presented are only for children and adolescents in Victoria and therefore cannot necessarily be applied to other states and territories or countries. It is generally assumed that all ACL injuries are sustained in sporting activity [5]. This study shows that the vast majority of cases in children and adolescents are associated with sport, but it is also possible that some injuries are associated with more informal activities such as general play and recreational activity in these age groups, rather than formal competitive sport. The circumstances leading to ACL injuries would be worthy of further epidemiological attention.

## 5. Conclusions

Despite the limitations, this paper provides new data that show a somewhat alarming increasing trend in ACL injury rates in children and adolescents over time. This study clearly demonstrates that ACL injuries in children and adolescents are a significant public health burden and this can have long-term effects on performance, participation, as well as physical and cognitive development and health. In the past, ACL injuries were believed to be only a problem for adults participating in competitive sports, but this study highlights such injuries as an emerging issue in children and adolescents too.

Exercise-based injury prevention programs have been developed that have demonstrated to decrease the risk of ACL injury, but their widespread adoption has not yet been achieved. These now need to be implemented in schools, starting with pre-pubescent children [2]. Further, junior sports teams at all levels should do the same [2].

Government health agencies, sports medicine authorities, and sporting bodies need to recognize ACL injury in children and adolescents as a priority issue, for both health and sustained participation in sport. Cost effective prevention programs are now available for implementation at a national level [48]. Given the increasing trends reported in this paper, and the previously reported increasing trends in all forms of sports injury in children [27], it is perhaps now time to consider the application of more structural, political, and population-focused measures to this important public health issue. The incidence of ACL injuries will continue to increase if not addressed and therefore it is time to identify, implement, and support injury prevention policies and programs for ACL injuries in children.

## Figures and Tables

**Table 1:** Proportion of health interventions for ACL injuries amongst hospitalized 5- to 14-year-olds in Victoria, Australia 2005/06 to 2014/15.

Australian Classification of Health Interventions (ACHI) for Admitted ACL Injuries amongst Victorian Children and Adolescents	Number	%
Arthroscopic reconstruction of knee	106	33.1
Reconstruction of knee	87	27.2
Arthroscopic reconstruction of cruciate ligament of knee with repair of meniscus	55	17.2
Reconstruction of cruciate ligament of knee with repair of meniscus	18	5.6
Arthroscopic debridement of knee	9	2.8
Arthroscopy of knee	8	2.5
Arthroscopic menisectomy of knee with debridement, osteoplasty or chondroplasty	7	2.2
Allied health intervention, physiotherapy	5	1.6
Other surgical procedures *	25	7.8
Total	320	100

* Combination of all other categories with individual cell counts less than five; the majority (77.2%) of patients spent less than two days in hospital. The remainder (22.8%) spent two to seven days.

**Table 2:** Detailed activity for ACL hospital admissions for males and females age 5–14 years in Victoria, 2005–2015 *.

Activity	Male		Female		Total
	*n*	%	*n*	%	
Team ball sports	62	35.4	76	52.4	138
Other activity (not specified)	83	47.4	46	31.7	129
Other specified activity with small cell counts grouped together (Team bat or stick sports, individual athletic activities, acrobatic sports, combative sports, equestrian activities, other school related recreational activities, other specified sport and exercise activity)	6	3.4	11	7.6	17
Other sport or leisure activities (Ice and snow sports, wheeled motor sports, wheeled non-motored sports, unspecified sport and exercise activity, leisure activity)	24	13.7	12	8.3	36
Totals	175	100	145	100	320

* Cell values containing counts of one to four cases have been suppressed.
